# Designing architecture of soothing labor–delivery–recovery–postpartum unit: a study protocol

**DOI:** 10.1186/s12978-020-01055-x

**Published:** 2020-12-10

**Authors:** Behnam Kazemi Esfeh, Ashraf Kazemi, Aida Shamsaie

**Affiliations:** 1 Faculty of Engineering, University of Kashan, Kashan, Iran; 2 Reproductive Health Department, Nursing and Midwifery Care Research Center, School of Nursing and Midwifery, Isfahan University of Medical Sciences, Hezarjerib AV, Isfahan, Iran; 3 Scene Design Department, School of Cinema and Theater, University of Art, Tehran, Iran

**Keywords:** LDRP, Architecture, Childbirth, Soothing

## Abstract

**Background:**

The physical environment profoundly affects women's well-being during childbirth in family-centered unit. A LDRP, which is an acronym for labor, delivery, recovery, and postpartum that describes a maternity unit designed for family-centered care. This study aimed to design soothing LDRP healing architecture based on recognizing the emotional elements of Iranian women.

**Methods:**

This study will be conducted in four consecutive phases; including review of literature to provide documentation based on architectural knowledge and the effects of each architectural component on the parturient psychological conditions, qualitative study to extract women's needs and suggestions for physiological childbirth, architectural designing of the soothing LDRP based on the results of the first and second phases and qualitative study for evaluation of the designed program.

**Discussion:**

Designing soothing LDRP architecture based on safe child birth unit standards and favorable psychological environment will provide a positive childbirth experience for Iranian women and their family. But, the preferences and demands of families will be based on Iranian socio-cultural context, therefore the using of this design will be limited in other societies with different cultures.

## Background

Childbirth is one of the most important experiences of women in reproductive age that, under the influence of its negative experiences, can be harmful and have negative effects on the women's mental health [1] and family health in general [2]. Anxiety and stress during childbirth are associated with an increase in the need for sedatives during labor [3], a decrease in the progression of uterine contractions and prolonged labor [4], and low APGAR score in the newborn [5]. Moreover, childbirth stress is associated with an increased risk of postpartum depression [6], disruption of the oxytocinergic system, followed by impaired mother–child attachment [7] and delayed breastfeeding.

Physical environment/setting is one of the factors affecting women's experience of childbirth. The physical environment profoundly affects women's experiences, health and well-being during childbirth. The introduction of design elements and strategies with the function of calming and reducing negative emotions can lead to positive experiences for women during childbirth [8]. Places and locations which are different from the normal living conditions of a person may hinder the feelings of peace and calmness. However, most of the environments where childbirth takes place are equipped based on a medical perspective on childbirth to provide medical care, and the entry of women into these unknown environments can be associated with anxiety and stress for them [8]. The reason is that these places are equipped with different medical equipment which may suggest a health-threatening condition in one's mind exacerbating the anxiety and stress of the parturient. Childbirth is a physiological process, and prenatal, during childbirth, postpartum and neonatal care is considered a normal life event accompanied by dynamic emotional, social and physical changes. However, childbirth in an unknown environment that is different from normal living conditions can change this attitude in women and turn it into an event with a therapeutic approach that is not free of anxiety [9].

A study in this regard has described childbirth in an environment with processed technology as being in a without-support, unsuitable environment [10]. Another study has referred to women's experience of childbirth as fear, anxiety, disability, and the imminent feeling of death [9]. Women's descriptions of the psychological harms of childbirth have also been reported as perceived threats, prioritization of medical prescriptions over parturient needs, and a sense of being ignored [11]. According to a qualitative study, the lack of family support during childbirth was a harmful factor for women's mental health during childbirth [12].

On the other hand, the potential of turning a physiological labor/childbirth into a high-risk one justifies the need for quick access to medical procedures during childbirth. In this regard, the design of the delivery room in a regional hospital in Sweden has been designed based on the healing architecture. The effect of the building and interior design of this hospital on the senses, including the experience of users' pain, has been considered in the architectural design of this center. Evaluation of the childbirth experience of women in this hospital has shown that its facilities comply with the needs of women in labor [8]. An interventional study showed that the simulation of home conditions, such as using natural landscapes and watching television on LDR during labor pains, while improving fetal heart rate and newborn APGAR score, reduces the need for painkillers during the childbirth and leads to the positive view of the mother [13].

Another interventional study showed that listening to music reduces labor pain and anxiety of women during childbirth [14]. Irmawati et al. reported that hearing the voice of Qur'an by Muslim women during childbirth reduced anxiety and blood cortisol levels in women [15]. These results indicate favorable maternal and neonatal outcomes in home birth compatible with normal conditions [16]. Moreover, in the provision of various health services, family-orientated care has been associated with the improvement of family relationships [17, 18] and their mental health [19]. In this regard, in hospitals and maternity centers of many countries, a unit has been considered under the abbreviated title of LDRP; which is an acronym for labor, delivery, recovery, and postpartum that describes a maternity unit designed for family-centered care. Women in labor and their family complete normal childbearing experiences in one homelike room and the newborn remain at the bedside throughout the stay.

The design of this unit is based on the view that childbirth is a natural phenomenon and in this unit the parturient and her family experience childbirth in a home-like setting; at the same time, they can utilize standard midwifery services and in case of childbirth complications, have access to specialized services. However, in LDRP design of hospitals in many countries, pragmatism is a major part of LDRP design thinking; so that in its design, little attention has been paid to the quality of the environment, based on scientific documents and cultural attitudes. Therefore, given the role of culture in the desires and motivations of individuals, the aim of the present study is to design LDRP healing architecture based on recognizing the emotional elements of Iranian women.

## Methods

This study approved by the Ethics Committee of Isfahan University of Medical Sciences in Isfahan, Iran (IR.MUI.RESEARCH.REC.1399.453), and will be conducted in Isfahan-Iran in three consecutive phases. The study phases include review of literature, qualitative study, architectural designing of the soothing LERP based on the results of the first and second phases, and evaluation of the program.

### Phase I: Review of literature

The aim of the study in this phase is to provide documentation based on architectural knowledge and the effects of each architectural component on the psychological conditions of people, focused on parturient. At this stage, extensive search will be carried out in Persian and English Electronic and manual databases in the period from 1990 to 2020. There will also be searched for the Iranian culture of childbirth. The existing standards for LDRP unit will also be considered.

### Phase II: Qualitative study

At this stage, the qualitative study will be conducted for extracting women's needs and suggestions for physiological childbirth in LDRP, using semi-structural interviews.

The setting of this phase of the research will be centers for providing prenatal and post-natal services in Isfahan. All of the interviews will be conducted in a private place selected based on the preference of the participants. The midwifes will also have interviewed in their workplaces. After analyzing the qualitative data by the content analysis method,

#### Participants

The participants of the qualitative phase of this research consist of four groups. Women who have had a history of labor in LDRP, women who have uncomplicated pregnancies and attend childbirth preparation classes and their husbands, and also, providers of delivery services (midwives). The participants will be selected using purposeful sampling method and considering the diversity of the age, education, number of parity. After evaluating their inclusion criteria and obtaining their informed consent, the participants will be interviewed. The interviews will be continued until the repetition of the data and the feeling of reaching data saturation.

#### Inclusion and exclusion criteria

In the qualitative study, Iranian women that a maximum of six months have passed from their delivery will be included in the study.

#### Qualitative data collection method

In the qualitative phase of the research, the data will be collected using in-depth interviews. To observe ethical considerations, the participants will be explained about the aims of the study and their informed consent will be obtained for recoding their voice. The setting and the length of the interviews will be specified based on the preferences of the participants. Data analysis will be performed using the conventional content analysis. To assure the validity and reliability of the findings of the research, the four criteria of credibility, dependability, transferability and confirmability will be considered [20].

### Phase III: Architectural designing of the soothing LDRP

In this phase of the study, the priorities of the program will be developed based on the results of the first and second phase. Using review of the literature, the initial draft of the program will be developed and its render will be captured and the validity of the program will be evaluated through using expert panel.

#### Holding a experts panel

An expert panel will be used for validating the program and its render. The members of the panel will consist of midwifery specialists, architect, and psychologists. Prior to the formation of the expert panel, the electronic version of the program (and the hard copy if needed), will be sent to the members of the panel. At the time of expert panel formation, the opinions of experts will be collected regarding the architectural capabilities designed to comply with midwifery standards and architectural standards for safe childbirth. Consensus of the experts of the panel will be considered in the design.

### Phase IV: Evaluation of the program

After the implementation of the project in the maternity ward of the Beheshti hospitals affiliated to Isfahan University of Medical Sciences, evaluation will be performed. For this regard a qualitative study will be conducted on women's birth experiences in the designed soothing LERP after execution of the plan.

#### Participants

The participants of evaluation phase consist of three groups. Women who will childbirth in the designed soothing LDRP and their husbands, and midwives. After obtaining their informed consent, the participants will be interviewed. The interviews will be continued until the repetition of the data and the feeling of reaching data saturation.

#### Data collection

The data will be collected using semi structural interviews. All ethical considerations, will be considered. Data will be analyzed using the conventional content analysis.

## Discussion

The aim of this study is to design a soothing LDRP architecture in which safe childbirth conditions are provided for women and their families in a favorable psychological environment and provide them with a positive experience. Childbirth is a painful process and, thus, creating a psychological atmosphere that relieves this pain as much as possible can provide women with a positive experience of childbirth. In LDRP architectural design, the choice of architectural components provides a calm psychological atmosphere which can be relaxing and increase the threshold of pain stimulation.

Accordingly, in the design of soothing LDRP architecture in this research, architectural components such as color, furniture, and lighting are designed based on scientific documents where these components have been shown to be soothing and relaxing. Additionally, the aim of this study is to design the soothing LDRP architecture based on the culture and psychological needs of the target group using the experiences and preferences of women and their families. Finally, safe labor requires the use of technology in emergencies, and the use of obstetricians provides the assurance that emergency care will be provided.

But this design will have limited use for other societies. Because the appropriate environment for childbirth is defined based on social and cultural preferences and cultural differences affect people's preferences.

## Data Availability

Not applicable.

## Abbreviation

LDRP: Labor, delivery, recovery, and postpartum
